# What Factors Are Associated With Patients Being Active Partners in the Management Fibromyalgia? A Mixed Methods Systematic Review Protocol

**DOI:** 10.1002/msc.70033

**Published:** 2024-12-23

**Authors:** Jessica Coggins, Mwidimi Ndosi, Jennifer Pearson

**Affiliations:** ^1^ School of Health and Social Wellbeing University of the West of England Bristol UK; ^2^ Academic Rheumatology Unit University Hospitals Bristol and Weston NHS Foundation Trust Bristol Royal Infirmary Bristol UK; ^3^ The RNHRD and Brownsword Therapies Centre Royal United Hospitals Bath NHS Foundation Trust Royal United Hospital Bath UK

**Keywords:** activation, active partner, engagement, fibromyalgia, fibromyalgia management, participation, systematic review

## Abstract

**Background:**

Fibromyalgia Syndrome (FMS) is characterised by widespread and persistent pain, intrusive fatigue and cognitive issues, affecting approximately 5.4% of the UK population. Non‐pharmacological therapies and education are current management recommendations, but these approaches rely on patients having an active role in their healthcare management. It is therefore important to identify the factors associated with FMS patients being active partners, as this could influence person‐centred care provision.

**Aim:**

The aim of this study is to explore the factors associated with patients being an active partner in the management of FMS.

**Methods:**

This is a protocol for mixed methods systematic literature review with convergent integrated approach in accordance with JBI methodology. The databases AMED, MEDLINE, PsychINFO and CINAHL will be searched via EBSCOhost. Screening and selection will be conducted by two reviewers. Primary qualitative, observational and experimental studies from July 2005 to July 2024 will be included. Critical appraisal of eligible studies will be conducted using appropriate JBI tools. Data will be extracted, transformed where necessary and synthesised without meta‐analysis.

**Discussion:**

This mixed methods systematic review will provide a comprehensive understanding of the factors associated with patients being active partners, offering not only the ‘what’ but also the ‘why’ behind patients taking an active role in their healthcare. This will help guide future research and practice in supporting patients to be active partners in FMS management.

**Trial Registration:**

This systematic review has been registered with PROSPERO (registration number: CRD42024575159)

## 1 Introduction

Fibromyalgia syndrome (FMS) is a complex, long‐term condition which affects approximately 5.4% of the UK population and 2.7% of the global population (Fayaz et al. 2016; Queiroz 2013). The syndrome is commonly characterised by nociplastic, widespread and persistent pain alongside other associated symptoms including sleep disturbances, intrusive fatigue, psychological distress and impaired physical and cognitive function (Royal College of Physicians 2022). The risk factors include being female, aged over 50, having pre‐existing medical conditions, smoking, a high body mass index, and lower socioeconomic status (Creed 2020). The aetiology and pathophysiology remain unknown, but abnormal pain processing within the central nervous system is the primary proposal (Clauw et al. 2018). Yavne et al. (2018) reported a significant association between prior physical or psychological trauma and the subsequent development of FMS; however, the condition can also develop in the absence of trauma.

Current FMS management recommendations include non‐pharmacological therapies and patient education. Non‐pharmacological therapies include aerobic and strengthening exercise, multicomponent therapies, defined physical therapies (acupuncture or hydrotherapy), meditative movement therapies and mindfulness‐based stress reduction (Macfarlane et al. 2017). In rheumatology, patient education interventions include structured educational programmes about understanding the disease and treatment options, self‐management skills, and lifestyle modification (Zangi et al. 2015). The management of FMS should ensure to include shared decision‐making between healthcare professionals and patients, as this helps patients to feel better informed, well supported and more satisfied with care provision (Doebl, Macfarlane, and Hollick 2020; Macfarlane et al. 2017).

Due to the premise of non‐pharmacological therapies and patient education, it is important that patients with FMS play an active role in their healthcare management. Multiple interconnected concepts are used to describe a patient's active role in their healthcare, including empowerment, activation, engagement, enablement, involvement, participation and adherence (Hickmann, Richter, and Schlieter 2022). However, these related concepts are often used interchangeably within research and clinical practice as they are not well defined in the literature, resulting in a lack of understanding and poor communication (Hickmann, Richter, and Schlieter 2022; Jiang, Kong, and Jiang 2017).

For the purposes of this review, the term ‘active partner’ will be utilised to describe individuals who become co‐managers of their own health by demonstrating high levels of the stated concepts as described in the systematic literature review by Hickmann, Richter, and Schlieter. (2022). A patient as an active partner is an individual who is actively involved in their own care and decision‐making processes. Behavioural examples could include seeking information, participating in shared decision making, following agreed plans and recommendations, engaging in collaborative relationships with healthcare providers and having open communication. A collaborative partnership between healthcare providers and patients is necessary to improve health outcomes, increase patient and provider satisfaction, create high performing and cost‐efficient systems, and lead to effective resource allocation (Dwarswaard et al. 2016; Hickmann, Richter, and Schlieter 2022; National Health Service England 2017).

Previous work has been conducted to explore factors associated with singular concepts, such as patient activation and inflammatory arthritis (Jones et al. 2021), patient empowerment and rheumatoid arthritis (Larsson, Bremander, and Andersson 2021) and patient engagement and rheumatoid arthritis (Tan et al. 2019). However, factors associated with individuals being active partners in their healthcare have not been explored using multiple concepts and in the context of FMS. Thus, the aim of this study was to explore what is known about the factors that are associated with individuals living with FMS being active partners in their healthcare management. Understanding these factors could influence the way in which person‐centred care is provided (Themelis and Tang 2023).

## 2 Methods

### 2.1 Study Design and Registration

The protocol for this systematic literature review has been registered with the International Prospective Register of Systematic Reviews (PROSPERO; registration number: CRD42024575159). This review will follow the methodology and guidance of JBI (Lizarondo et al. 2024; Stern et al. 2020). A convergent integrated approach will be utilised as the research question can be answered using both quantitative and qualitative evidence and synthesis can occur simultaneously. Qualitisation through data transformation may be used to combine quantitative and qualitative findings.

### 2.2 Review Question

What factors are associated with patients being active partners in the management of FMS?

### 2.3 Eligibility Criteria

Population: This review will consider evidence that includes adults of all ages or genders with a diagnosis of FMS.

Phenomena of interest: Studies that investigate or explore any factors associated with patient activation, empowerment, engagement, enablement, involvement, participation or adherence will be considered.

Context: No limits will be used for geographical location or setting. Primary care, acute care, secondary care or tertiary care will all be eligible.

Types of studies: This review will consider qualitative, quantitative (both observational and experimental) and mixed methods primary studies. Mixed methods studies will only be considered if the data from both components (quantitative and qualitative) can be clearly extracted. Studies published in English peer‐reviewed journals from July 2005 to July 2024 will be included.

If any of the information detailed in the inclusion criteria is not clear in the published report, the authors will be contacted for clarification. The inclusion and exclusion criteria are displayed in Table 1.

**TABLE 1:** Inclusion and exclusion criteria.

Inclusion criteria	Exclusion criteria
Primary research	Protocols
Adult population	Secondary research (systematic literature reviews, scoping reviews, evidence synthesis)
Dates (from 2005)	Papers not in English
Fibromyalgia diagnosis	Book chapters
	Studies which include children
	Unconfirmed diagnosis
	Studies reviewing research or policy making participation

### 2.4 Search Strategy

Search terms were initially developed by identifying key concepts and related keywords relevant to the research question using the Participant‐Exposure‐Outcome (PEO) framework (Table 2). An initial scoping search of MEDLINE via EBSCOhost was conducted to help develop the full search strategy. Patient and public involvement contributors and clinicians were also consulted to identify further keywords. Table 3 presents the full search strategy for MEDLINE. The search will be restricted to adult population and articles published in English between July 2005 and July 2024. Searches will be re‐run prior to the final analysis to identify any further studies. The search strategy aims to identify published literature relating to factors associated with being an active partner in FMS.

**TABLE 2:** PEO framework.

Population	Adults with fibromyalgia
Exposure	Associated (explanatory, predictors, correlates) factors
Outcomes	Patient activation
	Patient empowerment
	Patient engagement
	Patient enablement
	Patient involvement
	Patient participation
	Adherence

**TABLE 3:** Pilot search strategy for MEDLINE (via EBSCO).

#1	Population	Patient
#2	Concept	Empower* OR activat* OR engage* OR enabl* OR involv* OR participa* OR centred* OR orientation OR self‐manag* OR self‐care OR shared decision making OR adherence OR drop out
#3	Context	Fibromyalgia
#4	Boolean terms	AND, OR
#5	Search string	#1 AND #2 AND #3
	Filters used	Adults only
		1st July 2004 to 31st July 2024
		English language

### 2.5 Information Sources

A thorough search of AMED, CINAHL Plus, MEDLINE and PsychINFO will be conducted via EBSCOhost. The reference list of all selected for critical appraisal will be utilised for forward citation searching. Searches will be re‐run prior to the final analysis to identify any further studies.

#### 2.5.1 Study Selection

Once the search has been completed, all identified records will be imported into the online platform Rayyan (https://new.rayyan.ai/) and duplicates removed automatically if there is a 98% match, or manually by one reviewer. The number of duplications will be recorded. Two independent reviewers will screen the title and abstract of each record, ensuring that the inclusion criteria are followed. The full text of those deemed eligible will be reviewed in full and assessed in detail against the inclusion criteria. All disagreements on eligibility at both stages will be resolved through discussion. If a solution is not achieved, a third reviewer will be consulted to support the final decision. Reasons for exclusion of full texts will be documented and reported in accordance with Preferred Reporting Items for Systematic Reviews and Meta‐analyses (PRISMA) diagram (Shamseer et al. 2015).

### 2.6 Assessment of Study Quality

Following study selection, all records that are eligible for inclusion in the systematic review will be assed for methodological quality. However, all eligible studies will be included regardless of the outcome. Each record will be assessed independently by two reviewers using the critical appraisal tools from JBI SUMARI. These will include checklists for qualitative research (Lockwood, Munn, and Porritt 2015), analytical cross‐sectional, cohort and case‐control (Moola et al. 2020), studies quasi‐experimental studies (Barker et al. 2024) and randomised controlled trials (Barker et al. 2023). Similar to the screening stage, any disagreements will be resolved through discussion between two reviewers, and if no resolution is reached, a third reviewer will be consulted. The results of the critical appraisal will be reported narratively in a table.

### 2.7 Data Extraction

A pre‐determined data extraction table will be created to support the extraction of both quantitative and qualitative data. The extraction table will include specific details about the methodology, populations, study methods and variables reported to be associated with being an active partner in the management of FMS.

In qualitative studies, only themes or subthemes that are relevant to the review will be extracted. Quotations will also be extracted where available and if they demonstrate relevance to the research question. A level of credibility will be assigned to each finding following the JBI guidance: not supported, credible and unequivocal (Lizarondo et al. 2024). Not supported data will be documented but not included in the synthesis.

The output of the quantitative analysis (inferential or descriptive statistic) will be extracted into the table, including both significant and non‐significant results. Data will be inputted into the extracted table verbatim from the primary study where possible. In the instance where this is insufficient to answer the review question, a narrative representation using other data from the study may also be included, but the reviewer will ensure that the extracted data is kept as close to the reported findings as possible. The approach used will be dependent on the eligible records.

### 2.8 Data Transformation

Once all data have been extracted and inputted into the table, all quantitative data will be transformed and ‘qualitised’ using textual descriptions and narrative interpretation, responding directly to the research question.

### 2.9 Data Synthesis and Integration

Synthesis without meta‐analysis will be carried out using a convergent integrated approach. Qualitative data and the qualitised data will be categorised based on the factors identified. The extracted and transformed evidence will be pooled together based on similarity to generate a set of integrated findings presented as statements. Convergence and divergence will be explored and described. Due to the qualitisation of the quantitative data, which will be synthesised narratively, heterogeneity will not be considered (Lizarondo et al. 2024).

### 2.10 Ethical Considerations

Due to the nature of the study (systematic literature review), ethical approval will not be required.

## 3 Conclusion

FMS is a long‐term condition, causing personal and societal impacts. Previous research has indicated the importance of non‐pharmacological therapies and self‐management strategies in the overall disease management and minimising the impact of the condition (Geraghty et al. 2021; Macfarlane et al. 2017). These can only be successful if individuals living with FMS are active in their healthcare management. As the management of FMS is reliant upon individuals living with FMS being able to be ‘active partners’, identifying the factors associated with this is extremely important.

The factors associated with patients being active partners are under‐explored in FMS. This systematic literature review presents a summary of available evidence, demonstrating which factors may be associated with patients being active partners in relation to FMS. This is likely going to inform the development of interventions aimed to support person‐centred care. It will contribute to enabling healthcare providers to provide person‐centre care in line with the biopsychosocial model (Bolton 2023; Themelis and Tang 2023).

Additionally, the findings may impact the way in which health care professionals' approach and understand how to support those living with FMS. Furthermore, interactions, treatments and self‐management strategies could be altered locally in response.

This systematic literature review may help identify gaps in the literature and inform the design of future research, such as exploring the perspectives of individuals with FMS and how specific factors facilitate or hinder their ability to be active partners in their care. Ultimately, this body of evidence will inform recommendations, including tools and resources, to empower individuals with FMS to take an active role in their healthcare management.

## Author Contribution

All authors were involved in the conception and design of the study, the development of the search strategy, and the drafting and finalisation of the manuscript.

## Conflicts of Interest

The authors declare no conflicts of interest.

## Data Availability

The authors have nothing to report.

## References

[msc70033-bib-0001] Barker, T. H. , N. Habibi , E. Aromataris , et al. 2024. “The Revised JBI Critical Appraisal Tool for the Assessment of Risk of Bias for Quasi‐Experimental Studies.” JBI Evidence Synthesis 22, no. 3: 378–388. 10.11124/JBIES-23-00268. 38287725

[msc70033-bib-0002] Barker, T. H. , J. C. Stone , K. Sears , et al. 2023. “The Revised JBI Critical Appraisal Tool for the Assessment of Risk of Bias for Randomized Controlled Trials.” JBI Evidence Synthesis 21, no. 3: 494–506. 10.11124/JBIES-22-00430. 36727247

[msc70033-bib-0003] Bolton, D. 2023. “A Revitalized Biopsychosocial Model: Core Theory, Research Paradigms, and Clinical Implications.” Psychological Medicine 53, no. 16. 10.1017/S0033291723002660. PMC10755226 37681273

[msc70033-bib-0004] Clauw, D. J. , Y. D’Arcy , K. Gebke , D. Semel , L. Pauer , and K. D. Jones . 2018. “Normalizing Fibromyalgia as a Chronic Illness.” Postgraduate Medicine 130, no. 1: 9–18. 10.1080/00325481.2018.1411743. 29256764

[msc70033-bib-0005] Creed, F. 2020. “A Review of the Incidence and Risk Factors for Fibromyalgia and Chronic Widespread Pain in Population‐Based Studies.” Pain 161, no. 6: 1169–1176. 10.1097/j.pain.0000000000001819. 32040078

[msc70033-bib-0006] Doebl, S. , G. J. Macfarlane , and R. J. Hollick . 2020. “‘no One Wants to Look After the Fibro Patient’. Understanding Models, and Patient Perspectives, of Care for Fibromyalgia: Reviews of Current Evidence.” Pain 161, no. 8: 1716–1725. 10.1097/j.pain.0000000000001870. 32701832

[msc70033-bib-0007] Dwarswaard, J. , E. J. M. Bakker , A. van Staa , and H. R. Boeije . 2016. “Self‐management Support From the Perspective of Patients With a Chronic Condition: A Thematic Synthesis of Qualitative Studies.” Health Expectations 19, no. 2. 10.1111/hex.12346. PMC5055271 25619975

[msc70033-bib-0008] Fayaz, A. , P. Croft , R. M. Langford , L. J. Donaldson , and G. T. Jones . 2016. “Prevalence of Chronic Pain in the UK: A Systematic Review and Meta‐Analysis of Population Studies.” BMJ Open 6, no. 6: e010364. 10.1136/bmjopen-2015-010364. PMC4932255 27324708

[msc70033-bib-0009] Geraghty, A. W. A. , E. Maund , D. Newell , et al. 2021. “Self‐management for Chronic Widespread Pain Including Fibromyalgia: A Systematic Review and Meta‐Analysis.” PLoS One 16, no. 7: e0254642. 10.1371/journal.pone.0254642. 34270606 PMC8284796

[msc70033-bib-0010] Hickmann, E. , P. Richter , and H. Schlieter . 2022. “All Together Now – Patient Engagement, Patient Empowerment, and Associated Terms in Personal Healthcare.” BMC Health Services Research 22, no. 1. 10.1186/s12913-022-08501-5. PMC9440506 36056354

[msc70033-bib-0011] Jiang, D. , W. Kong , and J. Jiang . 2017. “Patient Engagement in Randomized Controlled Tai Chi Clinical Trials Among the Chronically Ill.” Reviews on Recent Clinical Trials 12, no. 1. 10.2174/1574887111666160815105702. 27527892

[msc70033-bib-0012] Jones, B. , M. Ndosi , A. Hunt , D. Harcourt , and E. Dures . 2021. “Factors Associated With Patient Activation in Inflammatory Arthritis: A Multisite Cross‐Sectional Study.” Rheumatology Advances in Practice 5, no. Supplement_2: II35–II44. 10.1093/rap/rkab053. 34755027 PMC8570153

[msc70033-bib-0013] Larsson, I. , A. Bremander , and M. Andersson . 2021. “Patient Empowerment and Associations With Disease Activity and Pain‐Related and Lifestyle Factors in Patients With Rheumatoid Arthritis.” ACR Open Rheumatology 3, no. 12. 10.1002/acr2.11341. PMC8672186 34523815

[msc70033-bib-0014] Lizarondo, L. , C. Stern , J. Carrier , et al. 2024. “Chapter 8: Mixed Methods Systematic Reviews.” In JBI Manual for Evidence Synthesis, edited by E. Aromataris , C. Lockwood , K. Porritt , B. Pilla , and Z. Jordan , JBI. https://synthesismanual.jbi.global.

[msc70033-bib-0015] Lockwood, C. , Z. Munn , and K. Porritt . 2015. “Qualitative Research Synthesis: Methodological Guidance for Systematic Reviewers Utilizing Meta‐Aggregation.” International Journal of Evidence‐Based Healthcare 13, no. 3: 179–187. 10.1097/XEB.0000000000000062. 26262565

[msc70033-bib-0016] Macfarlane, G. J. , C. Kronisch , L. E. Dean , et al. 2017. “EULAR Revised Recommendations for the Management of Fibromyalgia.” Annals of the Rheumatic Diseases 76, no. 2: 318–328. 10.1136/annrheumdis-2016-209724. 27377815

[msc70033-bib-0017] Moola, S. , Z. Munn , C. Tufanaru , et al. 2020 In Systematic Reviews of Etiology and Risk, edited by E. Aromataris , C. Lockwood , K. Porritt , B. Pilla , and Z. Jordan , JBI Manual For Evidence Synthesis. JBI. https://synthesismanual.jbi.global.

[msc70033-bib-0018] National Health Service England . 2017. “Involving People in Their Own Health and Care.” https://www.england.nhs.uk/publication/involving‐people‐in‐their‐own‐health‐and‐care‐statutory‐guidance‐for‐clinical‐commissioning‐groups‐and‐nhs‐england/.

[msc70033-bib-0019] Queiroz, L. P. 2013. “Worldwide Epidemiology of Fibromyalgia Topical Collection on Fibromyalgia.” Current Pain and Headache Reports 17, no. 8. 10.1007/s11916-013-0356-5. 23801009

[msc70033-bib-0020] Royal College of Physicians . 2022. “The Diagnosis of Fibromyalgia Syndrome UK Clinical Guidelines.” www.rcp.ac.uk.

[msc70033-bib-0021] Shamseer, L. , D. Moher , M. Clarke , et al. 2015. “Preferred Reporting Items for Systematic Review and Meta‐Analysis Protocols (Prisma‐p) 2015: Elaboration and Explanation.” BMJ 349. 10.1136/bmj.g7647. 25555855

[msc70033-bib-0022] Stern, C. , L. Lizarondo , J. Carrier , et al. 2020. “Methodological Guidance for the Conduct of Mixed Methods Systematic Reviews.” JBI Evidence Synthesis 18, no. 10: 2108–2118. 10.11124/JBISRIR-D-19-00169. 32813460

[msc70033-bib-0023] Tan, X. L. , G. Pugh , F. Humby , and D. Morrissey . 2019. “Factors Associated With Physical Activity Engagement Among Adults With Rheumatoid Arthritis: A Cross‐sectional Study.” Musculoskeletal Care 17, no. 2. 10.1002/msc.1385. 30729653

[msc70033-bib-0024] Themelis, K. , and N. K. Y. Tang . 2023. “The Management of Chronic Pain: Re‐Centring Person‐Centred Care.” Journal of Clinical Medicine 12, no. 22. 10.3390/jcm12226957. PMC10672376 38002572

[msc70033-bib-0025] Yavne, Y. , D. Amital , A. Watad , S. Tiosano , and H. Amital . 2018. “A Systematic Review of Precipitating Physical and Psychological Traumatic Events in the Development of Fibromyalgia.” Seminars in Arthritis and Rheumatism 48, no. 1: 121–133. 10.1016/j.semarthrit.2017.12.011. 29428291

[msc70033-bib-0026] Zangi, H. A. , M. Ndosi , J. Adams , et al. 2015. “EULAR Recommendations for Patient Education for People With Inflammatory Arthritis.” Annals of the Rheumatic Diseases 74, no. 6. 10.1136/annrheumdis-2014-206807. 25735643

